# *Hsa_circ_0000285* contributes to gastric cancer progression by upregulating FN1 through the inhibition of miR‐1278

**DOI:** 10.1002/jcla.24475

**Published:** 2022-05-09

**Authors:** Xue Wang, Mao Tan, He Huang, Yanlei Zou, Mengqiao Wang

**Affiliations:** ^1^ Department of General Surgery Chengdu Fifth People’s Hospital Chengdu China

**Keywords:** circRNA, FN1, gastric cancer, hsa_circ_0000285, miR‐1278

## Abstract

**Background:**

Gastric cancer (GC) is one of the most severe cancers worldwide, particularly in China. Circular RNA (circRNA) plays an essential role in GC. *Hsa_circ_0000285* regulates the progression of several cancers. However, its role in GC has not been reported. This study elucidated the molecular mechanism and role of *hsa_circ_0000285* in GC progression.

**Methods:**

GC cells were transfected with silencers of *hsa_circ_0000285* and fibronectin 1 (*FN1*), an inhibitor of miR‐1278, and their negative controls (NC). Mice were injected with short hairpin (sh) RNAs targeting *hsa_circ_0000285* or NC. The expression levels of *hsa_circ_0000285*, miR‐1278, and *FN1* were assessed using western blotting and reverse transcription quantitative real‐time polymerase chain reaction (qRT‐PCR). Several assays were used to evaluate cell proliferation, invasion, and apoptosis. Tumor burden was also analyzed. The interactions between miR‐1278, *hsa_circ_0000285*, and *FN1* were ascertained using dual‐luciferase reporter assays. An RNA immunoprecipitation (RIP) assay was used to assess the enrichment of *hsa_circ_0000285* and miR‐1278 in GC.

**Results:**

*Hsa_circ_0000285* was significantly overexpressed in the GC tissues. Silencing *hsa_circ_0000285* inhibited cell proliferation and invasion, promoted apoptosis, and inhibited tumor development. *Hsa_circ_0000285* sponged miR‐1278. Inhibition of miR‐1278 *in vitro* reversed the effects of *hsa_circ_0000285* silencing on GC progression. MiR‐1278 targeted *FN1*, and silencing *FN1* neutralized the effects of miR‐1278 inhibitors on GC progression.

**Conclusions:**

The *hsa_circ_0000285*/miR‐1278/*FN1* axis regulated GC progression. In addition, it may serve as a potential therapeutic biomarker for GC.

## Graphical abstract

In gastric cancer (GC), circ_0000285 suppresses miR‐1278 via sponging effects, and miR‐1278 suppresses FN1 expression via binding to its 3’UTR. Circ_0000285 regulates the miR‐1278/FN1 axis to promote GC cell malignant behaviors in GC development.

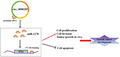

## 1 INTRODUCTION

Gastric cancer (GC) is one of the most common malignant tumors. In China, it is the second most common malignant tumor of the digestive tract.^1^ Its incidence significantly increases among people aged ≥50 years.^2^ In addition, its incidence among men is 2.4 times than that among women.^3^ Several factors, including environment, diet, infection, genetics, and immunity, are possible causes of GC.^2^ China has a high incidence of GC, and its annual prevalence and mortality rates are much higher than those of other countries.^4^ The 5 years relative survival rate is as low as 10% in patients diagnosed with advanced‐stage GC.^5^, ^6^ Metastasis is a significant cause of death in patients with GC. However, the process of invasion and metastasis in GC is complicated. Therefore, an in‐depth analysis of the molecular mechanisms underlying GC progression is necessary.

Circular RNA (circRNA) is a type of single‐stranded RNA that features a covalently closed ring structure without 5’ caps and 3’ poly (A) tails, and it can be divided into noncoding and coding circRNAs.^7^, ^8^ Several circRNAs have been identified recently using next‐generation sequencing.^9^ CircRNA is expressed in mammals and can influence the process of life through a complex regulatory network.^10^ CircRNAs can regulate the expression of microRNAs (miRNAs), thereby affecting the expression of miRNAs downstream of the target mRNA.^11^, ^12^ Therefore, circRNAs can influence the evolution, growth, and invasion of cells through circRNA/miRNA/mRNA regulatory mechanisms. This regulatory network helps classify histology, regulate disease progression, and explore strategies in GC.^13^, ^14^, ^15^ For example, the *hsa_circ_0004771*/miR‐149‐5p/AKT1/mTOR^16^ and CircPDSS1 (*hsa_circ_0093398*)/miR‐186‐5p/NEK2^17^ pathways have been shown to promote GC progression.

*Hsa_hsa_circ_0000285*, a circRNA generated from homeodomain interacting protein kinase 3 (HIPK3), suppresses the progression of various cancers, including cervical cancer,^18^ osteosarcoma,^19^, ^20^ thyroid cancer,^21^ and hepatocellular carcinoma.^22^ However, whether and how it affects GC has rarely been studied. In this study, we evaluated the regulation of *hsa_circ_0000285* in GC and investigated whether and how it influences GC via the circRNA/miRNA/mRNA axis. Our current study provides a new view of the function of *hsa_circ_0000285* in GC. In addition, a potential therapeutic target for GC has been discovered.

## 2 MATERIALS AND METHODS

### 2.1 Clinical samples and ethics statements

GC tissues (*n* = 40) and their corresponding normal adjacent tissues (*n* = 40) were collected from patients admitted to the Chengdu Fifth People's Hospital. The tissue specimens were quickly subjected to liquid nitrogen freezing and then kept at −80°C. None of the patients had received radiotherapy or chemotherapy prior to surgery. Histopathological evaluation was conducted to verify whether the patients had GC. Written informed consent was obtained from all the patients. This study was approved by the Ethics Committee of Chengdu Fifth People's Hospital, China. The human tissues involved in this study were handled appropriately and strictly in compliance with the standards of the Declaration of Helsinki.

### 2.2 Cell culture and transfection

The GC cell line and human gastric epithelial cell line GES‐1 were purchased from the American Type Culture Collection (ATCC). The HGC‐27, AGS, and GTL‐16 cell lines were inoculated into Dulbecco's modified Eagle's medium (DMEM; Gibco, Thermo Fisher Scientific, Inc.). The GES‐1 cells were cultured in a Roswell Park Memorial Institute (RPMI)‐1640 medium. All cell cultures were maintained at 37°C and 5% CO_2_. With the aid of a Lipofectamine™ 2000 Transfection Reagent (Thermo Fisher Scientific, Inc.), GTL‐16 and HGC‐27 cell lines were transfected for 6 h with silencers for *hsa_circ_0000285* (si‐circ) and fibronectin 1 (*FN1*; si‐FN1), an inhibitor of hsa‐miR‐1278, and their respective negative controls (NC) manufactured by GenePharma. Finally, qRT‐PCR analysis was used to evaluate the transfection efficiency 48 h post‐transfection.

### 2.3 Extraction of RNA and qRT‐PCR

TRIzol reagent (Invitrogen) was used to extract total RNA from clinical samples, GES‐1, HGC‐27, AGS, and GTL‐16 cell lines, and cells transfected with si‐circ, si‐FN1, si‐NC, inhibitor, inhibitor NC, and si‐*circ_0000285*+ miR‐1278 inhibitor (si‐circ+inhibitor). For 15 min, the extracted total RNAs (2 µg) was incubated at 37 °C with RNase R (Epicenter Biotechnologies, Shanghai, China). Table 1 lists the primers used, which were designed and synthesized by Sangon. To characterize the circRNAs, total RNA was digested by incubation at 37°C with RNase R (GeneSeed,). qRT‐PCR was conducted using miRNA Universal SYBR qPCR Master Mix (Cat#: MQ101) and HiScript II One Step qRT‐PCR SYBR Green Kit (Cat#: Q221‐01), provided by Vazyme Biotech Co. Ltd. Finally, *hsa_circ_0000285*, *FN1*, and miR‐1278 expression levels were determined using an Applied Biosystems (ABI) 7900HT Fast Real‐Time PCR System (Thermo Fisher Scientific, Inc.). The expression levels were normalized to those of *β*‐actin and calculated using the 2^−ΔΔCt^ method.

**TABLE 1:** Primer sequences for qRT‐PCR

Gene	Primer sequence (5’−3’)
circ_0000285	Forward primer: GCTCAGTTTGGTTGTGGTGA
	Reverse primer: TCACATGAATTTAGGTGGGACTT
miR−1278	Forward primer: GGCTCTGGCTCCGTGTCTT
	Reverse primer: CAGTGCAGGGTCCGAGGTATT
FN1	Forward primer: GTTCGGGAGGAGGTTGTTACC
	Reverse primer: GAGTCATCTGTAGGCTGGTTTAGG
U6	Forward primer: CAAATTCGTGAAGCGTTCCATA
	Reverse primer: AGTGCAGGGTCCGAGGTATTC
GAPDH	Forward primer: GGCTCATGACCACAGTCCATG
	Reverse primer: TCAGCTCTGGGATGACCTTG

### 2.4 Subcellular localization of *hsa_circ_0000285*

In accordance with the manufacturer's instructions, cytoplasmic and nuclear RNAs were extracted using the PARIS Kit (Thermo Fisher Scientific, Inc.; Cat#:AM1921). The distribution of *hsa_circ_0000285* within the nucleus and cytoplasm was assessed by qRT‐PCR and was normalized to *U6* and glyceraldehyde‐3‐phosphate dehydrogenase (*GAPDH*), respectively.

### 2.5 Evaluation of proliferation, invasion, and apoptosis

Cell proliferation, invasion, and apoptosis were evaluated to determine the functions of *hsa_circ_0000285*, miR‐1278, and *FN1* in GC progression.

Cell proliferation was evaluated using the cell counting kit‐8 (CCK‐8; Meilunbio, China). The transfected cells were inoculated into culture plates with 24 wells and then maintained for 24, 48, and 72 h. Then, each cell was supplemented with 10 μl of CCK‐8 reagent into the wells. The optical density (OD_450_) of the wells was measured at 450 nm using a BioTek microplate reader.

A Transwell assay was used to evaluate cell invasion. The upper Transwell sections were precoated with Matrigel and subsequently filled with a serum‐free medium before they were incubated at 37°C for 5 h. The bottom sections were carefully filled with 800 µL of an FBS‐containing (20%) medium and then incubated at 37°C for 24 h. The cells were washed, fixed, and stained with glutaraldehyde and 0.1% crystal violet. Finally, cells from five randomly selected views were observed and counted using a 400× microscope.

Flow cytometry was used to evaluate apoptosis. The Annexin V‐FITC/PI Apoptosis Detection Kit (Cat#: A211‐01; ) was obtained from Vazyme Co., Ltd. Transfected cells (1 × 10^5^) were collected and pre‐coated with phosphate buffered saline (PBS). The cells were subsequently washed with PBS (1 ml) and resuspended in (200 μl) binding buffer. Propidium iodide (PI; 200 μl) was used to stain the cells before they were incubated at 25°C for 30 min. Finally, a Becton Dickinson flow cytometer (BD FACScan) was used to assess cell viability.

### 2.6 Dual‐luciferase reporter experiment

The miR‐1278 complementary sites on *FN1* and *hsa_circ_0000285* were identified using StarBase (http://starbase.sysu.edu.cn) and Circular RNA Interactome (https://circinteractome.nia.nih.gov), respectively. In accordance with the binding sequences of miR‐1278, psiCheck‐2 vectors (Promega, Madison, WI, USA) were used to construct wild‐type (WT) and mutant (Mut) *hsa_circ_0000285* and *FN1* vectors: WT‐circ_0000285, Mut‐circ_0000285, WT‐FN1, and Mut‐FN1. With the aid of a Lipofectamine™ 2000 Transfection Reagent (Thermo Fisher Scientific Inc.), the constructed WT‐ and Mut‐ reporter vectors were transfected together with either a miR‐1278 mimic or a NC mimic into HGC‐27 and GTL‐16 cells. Transfection was conducted in accordance with the product protocol. At 48 h post‐transfection, luciferase activity was determined using a dual‐luciferase reporter assay system (Promega). Relative luciferase activity was normalized to that of *Renilla*.

### 2.7 RNA immunoprecipitation (RIP) assay

This experiment employed the Magna RIP RNA‐binding protein immunoprecipitation kit (Sigma‐Aldrich). HGC‐27 and GTL‐16 cells were disintegrated in lysis buffer and exposed to magnetic beads with anti‐IgG (NC) and Anti‐Ago2 coating. The RNAs attached to the beads were eluted and purified before they were evaluated using qRT‐PCR.

### 2.8 Western blotting

Radioimmunoprecipitation assay (RIPA) lysis buffer (Cat#: R0278, Sigma) was used for protein extraction. The proteins were quantified using a BCA kit (Solarbio). Western blotting was conducted as previously described.^23^ Standard sodium dodecyl sulfate‐polyacrylamide gel electrophoresis (SDS‐PAGE; 10%) was used to separate 80 μg of protein. The proteins on the gel were subsequently transferred onto polyvinylidene fluoride (PVDF) membranes. Skimmed milk (5%) was added to the membranes for blocking, which were then supplemented with primary antibodies such as anti‐*FN1* (Cat. no. ab2413; 1:1,000) and anti‐*GAPDH* (Cat. no. ab245357; 1:500). The primary antibodies were sourced from Abcam. The membranes were washed and incubated at 4℃ for 2 h with anti‐rabbit secondary antibodies (1:5,000, #AS014; ABclonal,). Finally, the PVDF membranes were washed for 1 min and subjected to an electrogenerated chemiluminescence (ECL) solution (ECL808‐25; Biomiga,). Bands were observed using a Tanon 6600 Luminescence Imaging Workstation (Tanon).

### 2.9 Tumor xenograft mice model

Sequences of small hairpin RNAs for *hsa_circ_0000285* (sh‐circ) and NC (sh‐NC) were designed and manufactured by GenePharma. The sh‐RNA was assembled into pSUPER‐retro‐puromycin plasmids (Cat#: VEC‐pRT‐0002; OligoEngine) and transfected into the HGC‐27 and GTL‐16 cells.

Ten 4‐week‐old BALB/c nude mice t (5 mice per group; Charles River,) were maintained at room temperature (25  ±  2°C), subjected to a 12 h light/dark cycle, and provided food and water *ad libitum*. Transfected cells were administered to the mice to induce tumor growth. Tumor volume was measured following the procedure documented by Qian et al.,^24^ wherein tumor data were collected weekly until the 5^th^ week post‐injection. After the experiment, the mice were euthanized in a CO_2_ chamber prior to tumor excision. The extracted tumors were photographed, weighed, and measured. This animal experiment was conducted in accordance with the ARRIVE guidelines and authorized by the Ethics Committee of Chengdu Fifth People's Hospital.

### 2.10 Statistical analysis

Each experiment was conducted in triplicate. GraphPad Prism 8 (San Diego, CA, USA) was used for statistical analysis and preparing graphs. Statistical tests included two‐way ANOVA (sub‐localization of *hsa_circ_0000285* research), unpaired Student's *t*‐test (comparison analysis), and Tukey's multiple comparison test, depending on the groups. Spearman's correlation analysis was used to evaluate the relationship between miR‐1278, *hsa_circ_0000285*, and *FN1*. Data are presented as mean ± standard deviation (SD). Differences were considered statistically significant at *p* < *0*.*05*.

## 3 RESULTS

### 3.1 *Hsa_circ_0000285* is significantly overexpressed in GC

**FIGURE 1:**
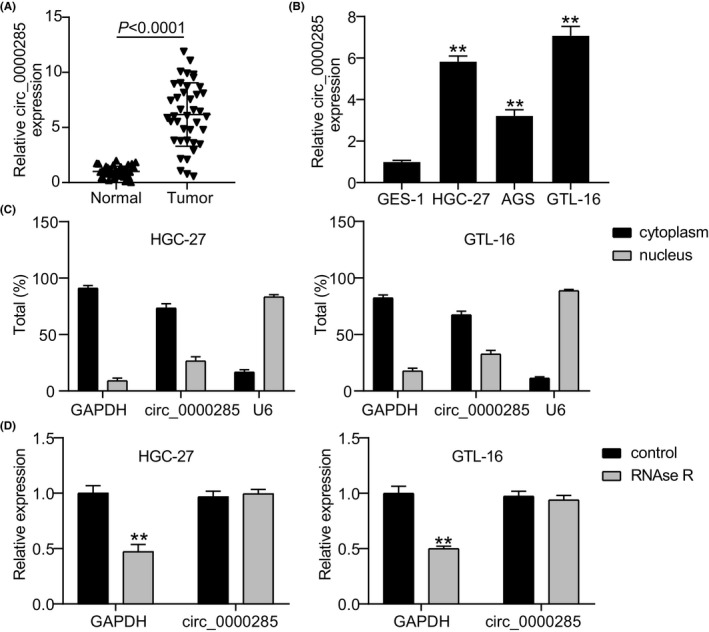
Overexpression of *circ_0000285* in GC. (A) The expression level of *hsa_circ_0000285* was evaluated in tumor tissues and normal tissues by qRT‐PCR. (B) QRT‐PCR was conducted to quantify the relative expression levels of *hsa_circ_0000285* in GES‐1, HGC‐27, AGS, GTL‐16 cells lines. ^**^ *p* < 0.001 *vs* GES‐1. (C) Relative *hsa_circ_0000285* levels were evaluated in the cytoplasm and nucleus by comparing with that of *GAPDH* and *U6*, respectively. (D) Relative *hsa_circ_0000285* and *GAPDH* expressions in HGC‐27 and GTL‐16 cells lines after RNase R digestion. ^**^ *p* < 0.001 *vs* control

*Hsa_circ_0000285* expression level in GC was evaluated and its expression level in tumors was found to be approximately six times that in normal tissues (*p* < 0.0001; Figure 1A). For the *in vitro* experiments, *hsa_circ_0000285* expression levels were assessed in AGS, GTL‐16, HGC‐27, and GES‐1 cell lines. qRT‐PCR results showed that *hsa_circ_0000285* was significantly overexpressed in the AGS (*p* < 0.001), GTL‐16 (*p* < 0.001), and HGC‐27 (*p* < 0.001) cells than in GES‐1 cells (Figure 1B). Therefore, we selected the HGC‐27 and GTL‐16 cell lines for subsequent experiments. Using qRT‐PCR, we assessed *hsa_circ_0000285* levels within the cytoplasm and nucleus, normalizing them against those of *GAPDH* and *U6*, respectively (Figure 1C). We found that *hsa_circ_0000285* was predominantly localized within the cytoplasm of both HGC‐27 and GTL‐16 cells, indicating that *hsa_circ_0000285* may function as a competing endogenous RNA (ceRNA) that plays a regulatory role in the development of GC. Additionally, the circRNA structure of *hsa_circ_0000285* was identified using RNase R. *Hsa_circ_0000285* exhibited better resistance to RNase R digestion than *GAPDH*, which has a linear structure (Figure 1D). This confirms the closed structure of *hsa_circ_0000285*. Overall, significant upregulation of *hsa_circ_0000285* was observed *in vitro* and in GC clinical samples.

### 3.2 Silencing *hsa_circ_0000285* retards the proliferative and invasive capabilities of cells, stimulates apoptosis, and inhibits tumor development

**FIGURE 2:**
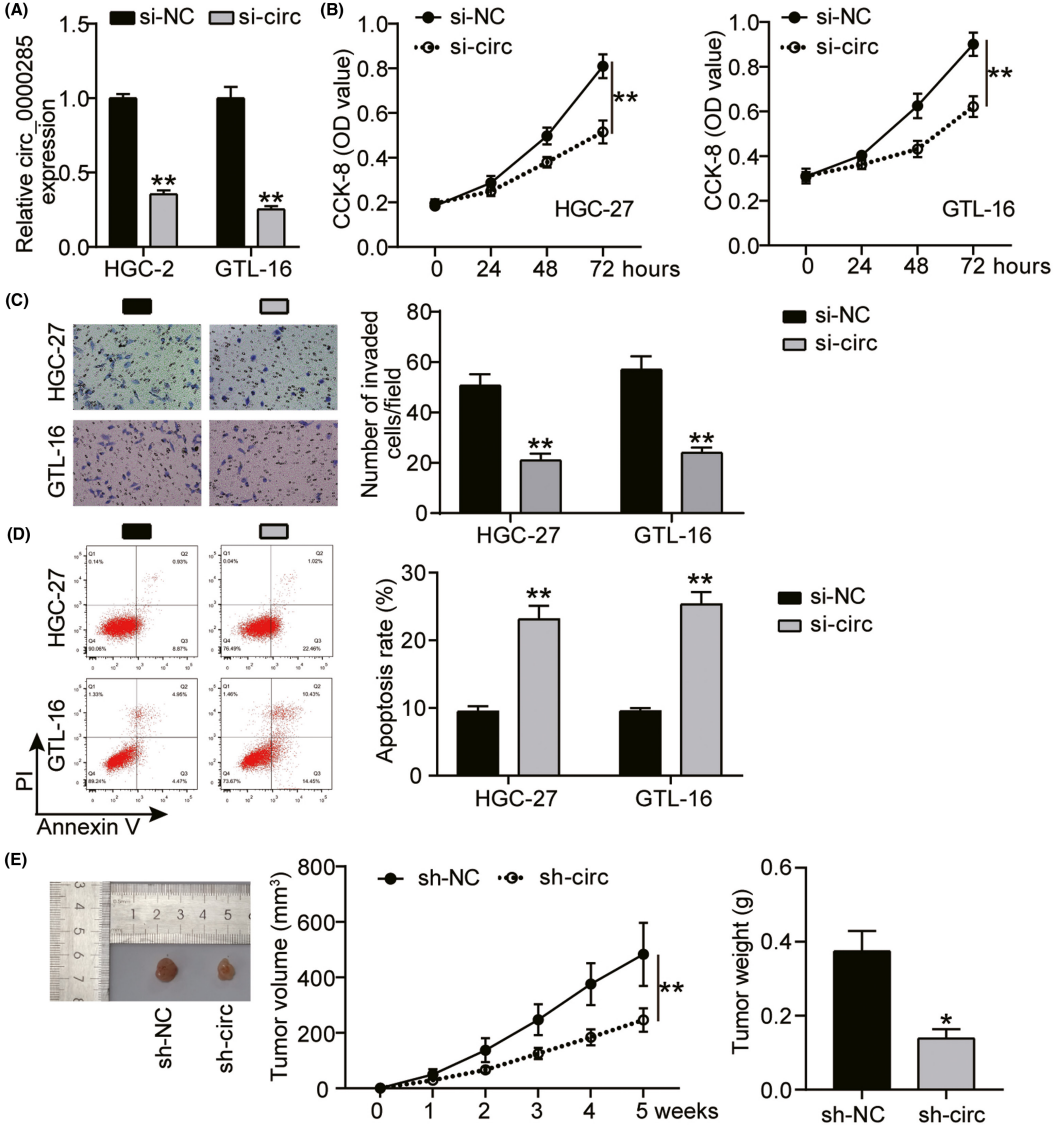
The function of silenced *hsa_circ_0000285* on GC progression. HGC‐27 and GTL‐16 cell lines were transfected with hsa_circ_0000285 silencers (si‐circ) and negative controls (si‐NC). (A) QRT‐PCR was carried out to evaluate transfection efficacy in the si‐ circ_0000285 and si‐NC group. ^**^ *p* < 0.001 *vs* si‐NC. (B) CCK‐8 assay was conducted to evaluate cell proliferation in the si‐circ_0000285 and si‐NC groups. ^**^ *p* < 0.001 *vs* si‐NC. (C) Transwell assay was performed to evaluate cell invasion in the si‐circ_0000285 and si‐NC groups. ^**^ *p* < 0.001 *vs* si‐NC. (D) Flow cytometry was conducted to evaluate the apoptotic rates in the si‐circ_0000285 and si‐NC groups. ^**^ *p* < 0.001 *vs* si‐NC. (E) Tumor volume and weight were measured among mice that were transfected with sh‐circ_0000285 and sh‐NC. ^*^ *p* < 0.05 *vs* sh‐NC

The role of *hsa_circ_0000285* in the regulation of proliferation, invasion, apoptosis, and tumor development was assessed. Transfection efficacy was ascertained by measuring *hsa_circ_0000285* expression levels in the si‐NC and si‐circ groups. qRT‐PCR analysis revealed downregulated *hsa_circ_0000285* expression after silencing, indicating successful transfection (*p* < 0.001; Figure 2A). In the HGC‐27 and GTL‐16 cell lines, cell proliferation was greatly repressed in the si‐circ group after 72 h of incubation compared to that in the si‐NC group (*p* < 0.001; Figure 2B). In addition, silencing *hsa_circ_0000285* significantly decreased the number of invasive HGC‐27 and GTL‐16 cells compared to that in the control group (*p* < 0.001; Figure 2C). Additionally, the apoptotic rate was higher in the si‐circ group than in the si‐NC group (Figure 2D). in the in vivo analysis, the mice in the sh‐circ group manifested smaller and lighter tumors than the mice in the sh‐NC group (Figure 2E). Altogether, in vivo and in vitro experiments showed that silencing *hsa_circ_0000285* suppressed the proliferation and invasion of cells, boosted apoptotic rates, and inhibited tumor development.

### 3.3 *Hsa_circ_0000285* sponges miR‐1278

**FIGURE 3:**
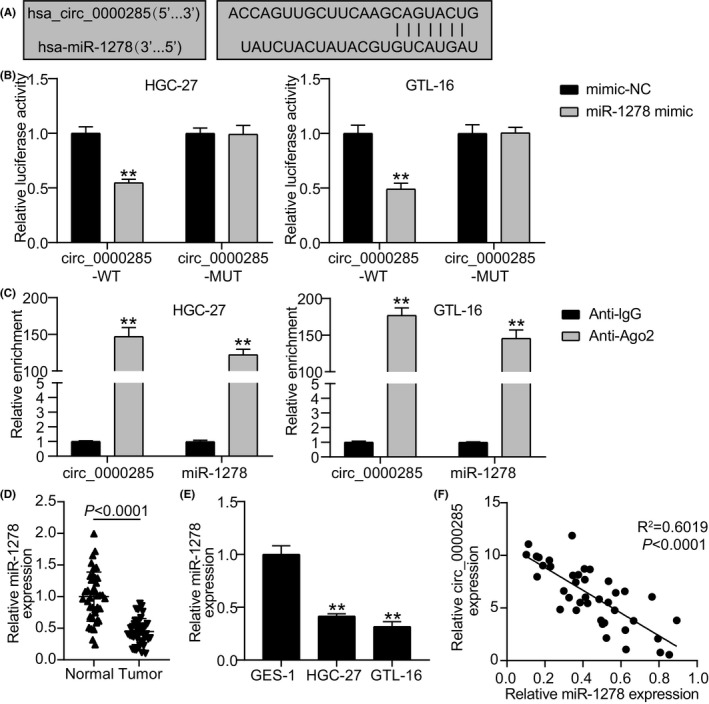
Sponging relationship between miR‐1278 and *hsa_circ_0000285*. (A) The binding sites of miR‐1278 on *hsa_circ_0000285* was predicted with the aid of Circular RNA Interactome. (B) Dual Luciferase‐reporter experiment confirmed miR‐1278 and *hsa_circ_0000285*’s association. ^**^ *p* < 0.001 *vs* mimic‐NC. (C) RIP assay confirmed the relationship between miR‐1278 and hsa_circ_0000285. ^**^ *p* < 0.001 *vs* Anti‐IgG. (D) Relative expression of miR‐1278 was evaluated in tumors and normal tissues of GC patients via qRT‐PCR. (E) QRT‐PCR was performed to evaluate the relative miR‐1278 expressions among the cell lines GES‐1, HGC‐27, and GTL‐16. ^**^ *p* < 0.001 *vs* GES‐1. (F) Correlation between miR‐1278 and hsa_circ_0000285 in tumor samples was assessed through Spearman's correlation

The abundance of *hsa_circ_0000285* in the cytoplasm enables it to serve as an miRNA sponge. Hence, its potential *hsa_circ_0000285* targets were identified using the Circular RNA interactome. Common binding sites were observed between miR‐1278 and *hsa_circ_0000285*, revealing a potential targeting relationship between the two (Figure 3A). Overexpression of miR‐1278 significantly decreased luciferase activity with WT‐circ_0000285 in the GTL‐16 and HGC‐27 cells (*p* < 0.001; Figure 3B). The enrichment of miR‐1278 and *hsa_circ_0000285* in the same complex further confirmed the relationship between miR‐1278 and *hsa_circ_0000285* (Figure 3C). These results verified that *hsa_circ_0000285* acts as an miR‐1278 sponge. The association between miR‐1278 and *hsa_circ_0000285* expression levels was investigated by measuring their expression in clinical samples and GC cell lines. It was observed that miR‐1278 was significantly downregulated in tumor samples and HGC‐27 and GTL‐16 cells, unlike in normal tissues and GES‐1 cells (Figure 3D,3E). Spearman's correlation analysis confirmed a negative correlation between miR‐1278 and *hsa_circ_0000285* expression (*R* ^2^ = 0.6019, *p* < 0.0001; Figure 3F). These findings confirm that *hsa_circ_0000285* sponges miR‐1278.

### 3.4 Inhibiting miR‐1278 reverses the suppressive effects of *hsa_circ_0000285* silencing on GC progression in vitro

**FIGURE 4:**
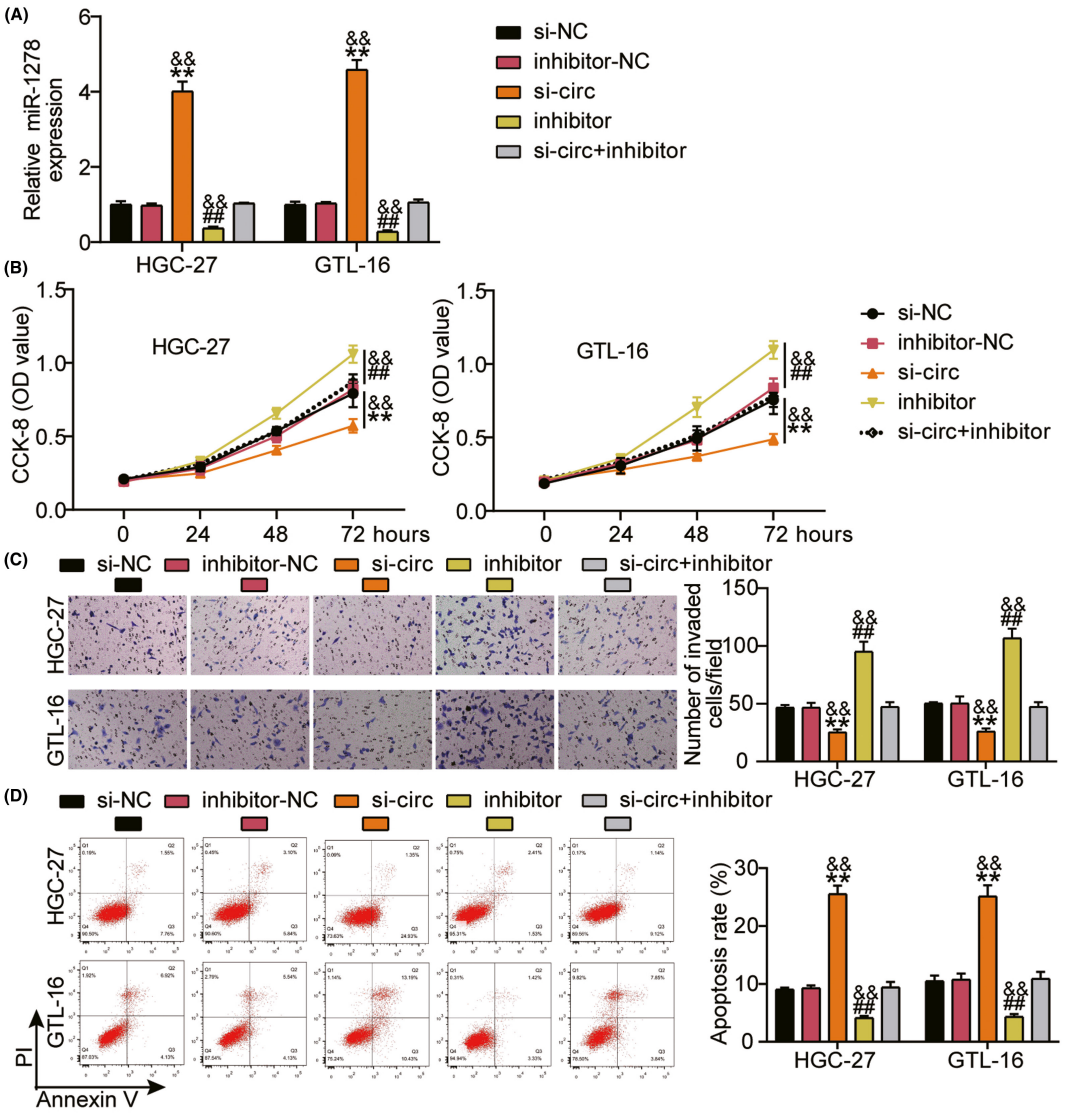
Inhibited miR‐1278 reverses the suppressive effects of *hsa_circ_0000285* silencing on the progression of GC *in vitro*. HGC‐27 and GTL‐16 cell lines were transfected with si‐NC, si‐circ_0000285 (si‐circ), inhibitor‐NC, miR‐1278 inhibitor (inhibitor), and si‐circ + inhibitor. (A) The expression of hsa_circ_0000285 was evaluated via qRT‐PCR in each group of cells. (B) Cell proliferation was assessed by conducting CCK‐8 assay in each group of cells. (C) Cell invasion was evaluated by Transwell assay in each group of cells. (D) Apoptosis was evaluated through flow cytometry assay in each group of cells. ^**^ *p* < 0.001 *vs* si‐NC; ^##^ *p* < 0.001 *vs* inhibitor‐NC; ^&&^ *p* < 0.001 *vs* si‐circ + inhibitor

The regulatory roles of miR‐1278 and *hsa_circ_0000285* in GC progression were also investigated. Figure 4A shows that miR‐1278 expression was remarkably upregulated after silencing *hsa_circ_0000285*, whereas it was downregulated in the presence of the miR‐1278 inhibitor. This revealed that the transfection of the silencers and inhibitors was successful. To study their effects on GC progression, cell proliferation, invasion, and apoptosis were assessed in the si‐NC, si‐*circ*, inhibitor‐NC, inhibitor, and si‐circ + inhibitor groups. Suppressing the expression of miR‐1278 significantly enhanced cell proliferation and invasion and retarded the apoptotic rate (*p* < 0.001; Figure 4B–D). The opposite was observed in the inhibitor‐NC group, but these outcomes were reversed after si‐circ transfection (*p* < 0.001; Figure 4B–D). These results suggested that MiR‐1278 and *hsa_circ_0000285* perform contradictory functions in the regulation of cell proliferation, invasion, and apoptosis. Hence, we concluded that silencing *hsa_circ_0000285* suppresses GC progression by increasing miR‐1278 expression.

### 3.5 MiR‐1278 targets FN1

**FIGURE 5:**
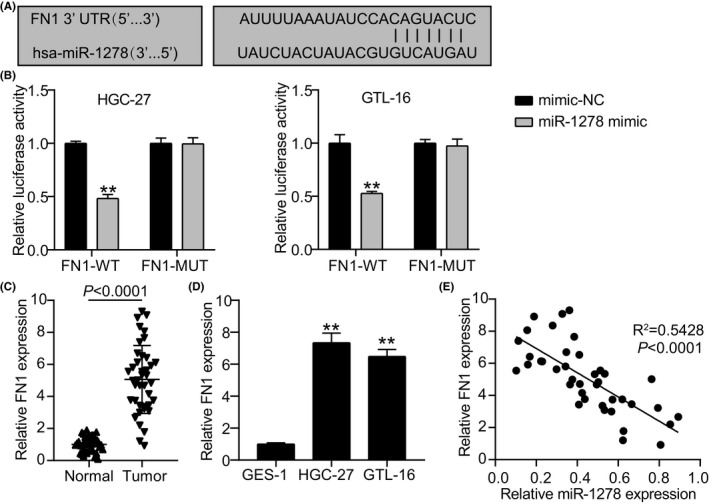
Relationship of miR‐1278 and FN1. (A) MiR‐1278’s binding sites on FN1 were predicted via StarBase. (B) Dual‐luciferase experiment confirmed the relationship between miR‐1278 and *FN1*. ^**^ *p* < 0.001 *vs* mimic‐NC. (C) The relative expression of FN1 was evaluated in tumors and normal tissues of GC patients via qRT‐PCR. (D) The expression level of FN1 was evaluated in cell lines of GES‐1, HGC‐27, and GTL‐16 through western blotting. ^**^ *p* < 0.001 *vs* GES‐1. (E) The correlation between miR‐1278 and *FN1* expressions in tumor samples was assessed using Spearman's correlation

StarBase predicted the binding sites of miR‐1278 on *FN1* (Figure 5A). Overexpression of miR‐1278 reduced the luciferase activity in WT‐FN1 by half (Figure 5B), but this inhibitory effect was absent in the Mut‐FN1 group. Hence, the dual‐luciferase assay confirmed the targeting relationship between miR‐1278 and *FN1*. Unlike normal tissues and GES‐1, *FN1* was significantly overexpressed in GC tumors and cells (Figure 5C and 5D). Furthermore, miR‐1278 and *FN1* expression were negatively correlated (*R* ^2^ = 0.5428, *p* < 0.0001; Figure 5E). These findings indicated that miR‐1278 targets *FN1*.

### 3.6 Silencing FN1 counteracts the effects caused by miR‐1278 inhibitor in the progression of GC in vitro

**FIGURE 6:**
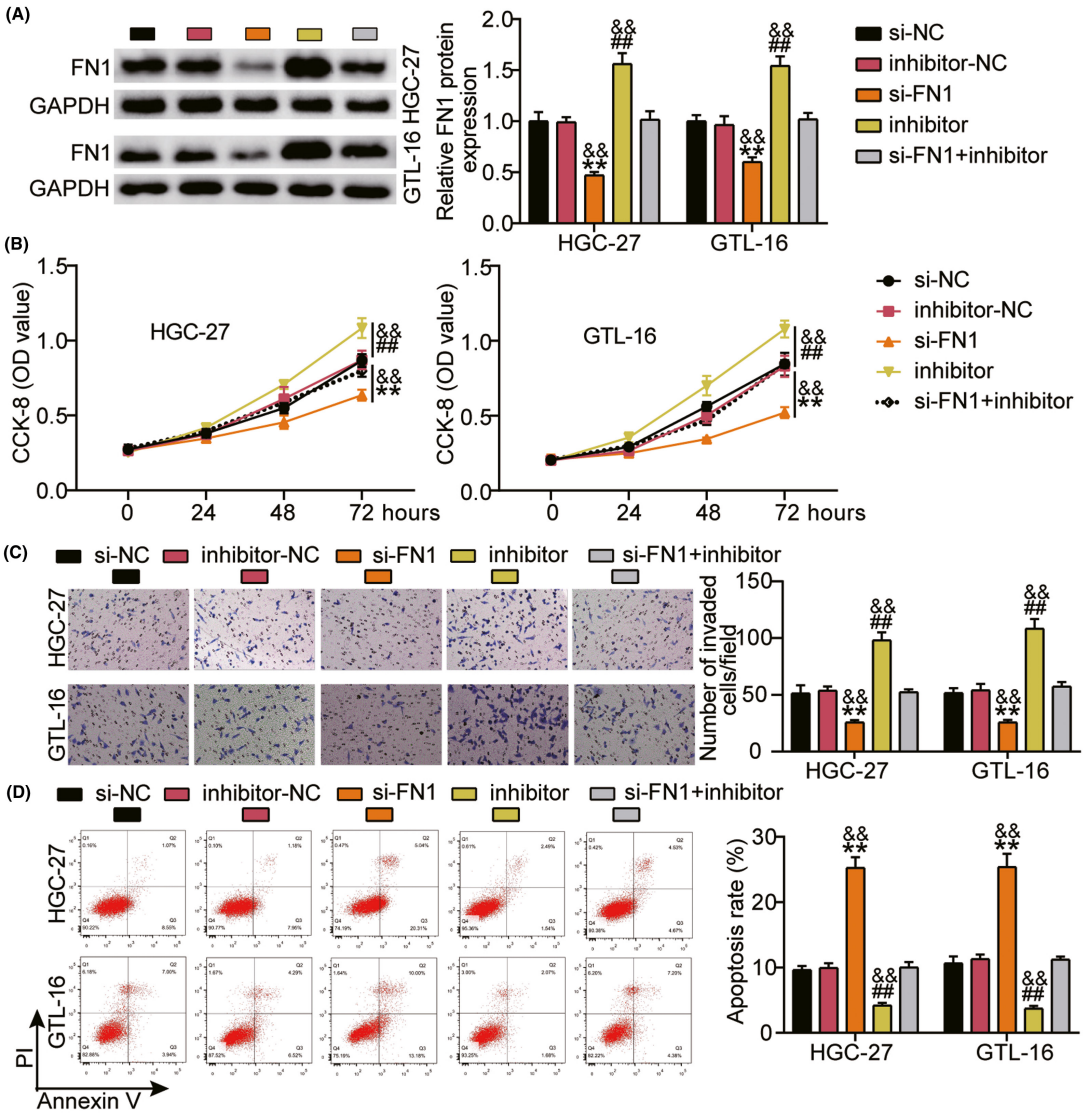
Silenced *FN1* counteracts the functions caused by miR‐1278 inhibitor on GC progression *in vitro*. HGC‐27 and GTL‐16 cell lines were transfected with si‐NC, si‐FN1, inhibitor‐NC, inhibitor, and si‐FN1+inhibitor. (A) The protein expression level of FN1 in each group of cells was quantified via western blotting. (B) CCK‐8 assay was conducted to evaluate the proliferation of cells in each group. (C) Transwell assay was performed to assess cell invasion in each group. (D) The apoptotic rate in each group was gauged through flow cytometry. ^**^ *p* < 0.001 *vs* si‐NC; ^##^ *p* < 0.001 *vs* inhibitor‐NC; ^&&^ *p* < 0.001 *vs* si‐FN1+inhibitor

The effects of silencing *FN1* and inhibiting miR‐1278 inhibition were investigated. First, the expression levels of *FN1* protein in the si‐NC, si‐FN1, inhibitor‐NC, inhibitor‐miR‐1278, and si‐FN1+inhibitor‐miR‐1278 groups were ascertained. A significantly downregulated FN1 expression level was observed in GTL‐16 and HGC‐27 cells with *FN1* knockdown. In addition, *FN1* was upregulated after the miR‐1278 expression was suppressed. *FN1* knockdown and miR‐1278 suppression had opposing effects on *FN1* expression (Figure 6A). Using in vitro cell function analysis, we discovered that silencing *FN1* inhibited the proliferative and invasive capabilities of the cells and stimulated apoptosis. This counteracted the effects induced by the miR‐1278 inhibitor on GC progression (Figure 6B–D), indicating that miR‐1278 represses GC progression by targeting FN1 in vitro.

## 4 DISCUSSION

An in‐depth understanding of the occurrence and progression of GC can be helpful for the development of GC therapeutics. CircRNAs are essential biomarkers for GC occurrence and progression. In this study, we explored the role of *hsa_circ_0000285* in the progression of GC. The results indicated that silencing *hsa_circ_0000285* suppressed the proliferation and invasion of cells, stimulated cell apoptosis, and inhibited tumor development. The sponging and targeting relationships between miR‐1278, *hsa_circ_0000285*, and *FN1* were also confirmed. This study revealed the function of *hsa_circ_0000285*/miR‐1278/*FN1* in the tumor growth, proliferation, invasion, and apoptosis of GC cells. Our findings elucidate a molecular mechanism in GC that may provide novel biomarkers for further improvement of GC therapeutics.

*Hsa_circ_0000285* was initially identified in bladder cancer and carcinomas. It serves as a prognostic biomarker, as it is correlated with tumor size, lymph node metastasis, TNM stage, and differentiation.^25^ *Hsa_circ_0000285* is overexpressed in several cancers, including osteosarcoma,^19^, ^20^ cervical cancer,^18^ thyroid cancer,^21^ and hepatocellular carcinoma.^22^ *Hsa_circ_0000285* regulates osteosarcoma progression by sponging miR‐409‐3p^19^ and hsa‐miRNA‐599.^20^ In thyroid cancer, *hsa_circ_0000285* sponges miR‐599 and enhances cell metastasis.^21^ These trends in circRNA expression in other cancers were consistent with those observed in our present study. When *hsa_circ_0000285* was silenced, cell proliferation and invasion were attenuated, apoptosis was enhanced, and tumor growth *in vivo* was suppressed. These findings regarding the functions of *hsa_circ_0000285* in GC are consistent with those in the aforementioned cancers. However, further research is required to explore the underlying mechanisms.

Considering the sponging relationship between circRNAs and miRNAs, the corresponding miRNAs sponged by *hsa_circ_0000285* were predicted. MiR‐1278 has been predicted to be sponged by *hsa_circ_0000285* in non‐small cell lung cancer,^26^ papillary thyroid carcinoma,^27^ and GC. Sponging miR‐1278 has been reported to produce contradictory effects in different cancers. For example, LINC00294 sponges miR‐1278 in glioma. This interaction enhances the levels of neurofilament medium (NEFM), a tumor suppressor in glioma, resulting in decreased cell proliferation.^28^ Similarly, in another study, miR‐1278 was sponged by circNEURL4 (*hsa_circ_0041821*), inhibiting the proliferation and invasion of papillary thyroid carcinoma.^27^ These findings suggest that circNEURL4 is a potential prognostic biomarker for papillary thyroid carcinoma.^27^ In contrast, Du et al.^26^ demonstrated that sponging of miR‐1278 by *hsa_circ_0101675* promoted non‐small cell lung cancer malignancy. Our study found that sponging of miR‐1278 by *hsa_circ_0000285* promoted the progression of GC. This is the first study to reveal the role of miR‐1278 in GC through sponging of *hsa_circ_0000285*.

The molecular mechanism underlying the role of miR‐1278 in GC progression was further investigated by predicting its target genes. We verified that *FN1* is a miR‐1278 target gene that counteracts the effects of miR‐1278 in GC progression. In this study, the targeting relationship between miR‐1278 and *FN1* was revealed for the first time. *FN1* is a member of the integrin receptor family that plays essential roles in the adhesion, growth, differentiation, and migration of cells by mediating the interaction between the extracellular matrix and cells.^29^, ^30^ *FN1* is involved in the progression of various cancers, such as esophageal squamous cell carcinoma,^31^ breast cancer,^32^ colorectal carcinogenesis,^33^ and nasopharyngeal carcinoma.^34^ A previous study confirmed that *FN1* knockdown could abate GC cell proliferation, migration, and invasion.^35^ It was also reported that *FN1* could act as a prognostic biomarker^36^, ^37^ and affect the clinicopathological parameters and prognosis of patients with GC.^38^ In this study, upregulation of *FN1* was observed in GC cell lines and tumors, which is consistent with the results of other studies.^35^, ^36^, ^37^, ^39^ We also demonstrated that silencing *FN1* repressed cell proliferation and invasion but boosted apoptosis. Considering all our findings, we conclude that *hsa_circ_0000285* contributes to the progression of GC by upregulating *FN1* through the inhibition of miR‐1278.

Our present study lacks validation from *in vivo* experiments. Therefore, further animal studies are necessary. Moreover, the number of clinical samples used in this study was insufficient. Considering the complexity of the molecular mechanisms, our present study cannot fully elucidate the role of the *hsa_circ_0000285*/miR‐1278/*FN1* axis in the progression of GC.

## 5 CONCLUSION

Silencing *hsa_circ_0000285* suppressed the proliferative and invasive capacities of GC cells, stimulated apoptosis, and impeded tumor development. In addition, the sponging and targeting relationships between miR‐1278, *hsa_circ_0000285*, and *FN1* were confirmed. Our work revealed the regulatory role of the *hsa_circ_0000285*/miR‐1278/*FN1* axis in GC progression. This axis may contribute to the identification of potential therapeutic targets in GC.

## AUTHOR CONTRIBUTIONS

MT and HH conducted the experiments and data analysis. XW devised and designed the study. YLZ obtained the data. XW and MQW performed the data analysis and interpretation. All authors read and approved the manuscript.

## CONFLICTS OF INTEREST

The authors declare that they have no conflicts of interest.

## ETHICAL APPROVAL

The present study was approved by the Ethics Committee of Chengdu Fifth People's Hospital (Chengdu, China). The processing of clinical tissue samples is in strict compliance with the ethical standards of the Declaration of Helsinki. All patients signed a written informed consent. All animal experiments were in strict compliance with the ARRIVE guidelines and were carried out in accordance with National Research Council's Guide for the Care and Use of Laboratory Animals.

## CONSENT TO PARTICIPATE

All patients provided a written informed consent.

## CONSENT FOR PUBLICATION

All participants provided consent for publication.

## Data Availability

The datasets that have been used and/or analyzed in this study are available from the corresponding author upon reasonable request.
